# Synthesis of intracellular and extracellular gold nanoparticles with a green machine and its antifungal activity

**DOI:** 10.3906/biy-2010-64

**Published:** 2021-04-20

**Authors:** Nurbanu GÜRSOY, Betül YILMAZ ÖZTÜRK, İlknur DAĞ

**Affiliations:** 1 Eskişehir Osmangazi University, Institute of Science, Biotechnology and Biosafety Department, Eskişehir Turkey; 2 Eskişehir Osmangazi University, Central Research Laboratory Application and Research Center, Eskişehir Turkey; 3 Vocational Health Services High School, Eskisehir Osmangazi University, Eskisehir Turkey

**Keywords:** *Chlorella*, green synthesis, gold nanoparticles, transmission electron microscope (TEM), antifungal

## Abstract

Green synthesis method is being increasingly used in the development of safe, stable, and eco-friendly nanostructures with biological resources. In this study, extracellular and intracellular synthesis of gold nanoparticles (AuNPs) was carried out using green algae *Chlorella sorokiniana* Shihira & R.W. Fresh algae were isolated and identified from Musaözü Pond located in the province of Eskişehir and then extraction process were performed. Optimization studies were studied using pH value, metal salt concentration, and time parameters for extracellular synthesis and using only time parameter for intrasellular synthesis. Since more controlled and optimum conditions can be achieved in the production of AuNPs by extracellular synthesis, these nanoparticles (NPs) were used for characterization and antifungal activity studies. Optical, physical, and chemical properties of synthesized NPs were characterized by UV visible spectrophotometer (UV-Vis), dynamic light scattering (DLS), Zetasizer, X-Ray diffraction (XRD), Fourier transform ınfrared spectroscopy (FTIR), field emission scanning electron microscope (FE-SEM), ınductively coupled plasma mass spectrometer (ICP-MS) and transmission electron microscope (TEM) analysis. The optimum conditions for AuNPs synthesis were determined as 1 mM for HauCl_4_ concentration, 6 for pH value, and 60th min for time. AuNPs obtained from extracellular synthesis from *C. sorokiniana* extract are 5–15 nm in size and spherical shape. TEM images of extracellular synthesis show noticeable cell wall and membrane damages, cytoplasma dissolutions, and irregularities. AuNPs obtained by intracellular synthesis are in 20–40 nm size and localized in the cell wall and cytoplasm. These NPs exhibited significant antifungal activity against *C. tropicalis*, *C. glabrata,* and *C. albicans* isolates. AuNPs obtained by algae-mediated green synthesis have a significant potential for medical and industrial use, and this eco-friendly synthesis method can be easily scaled for future studies.

## 1. Introduction

Metal nanoparticles (NPs) are widely used in optical, electronic, and biomedical sciences with their unique physical and chemical properties (Khan and Saeed, 2019). Although they can be synthesized by many different chemical methods, most of the initiators and reactants used in the reactions are toxic and potentially dangerous. In addition, undesirable by-products occurring during the process significantly limit the application possibilities and biocompatibility of the produced nanomaterial. The production of metal NPs with the green synthesis method can provide a low cost and eco-friendly method that can be renewed by reducing the use of unsafe chemicals and minimizing the occurrence of hazardous wastes (Kouvaris et al., 2012). Microorganisms, fungi, plants, or algae can be used for this method. When biological agents take metal ions from the environment, they convert elemental metal into nanoscale particles by enzymatic activities (Li et al., 2011). Biological resources can be used for nanoparticles synthesis either extracellularly or intracellularly, but intracellular synthesis are required additional purification steps. NPs produced by green synthesis have higher catalytic activity, have a greater specific surface area, and also have improved contact between the enzyme and the metal salt (Kalabegishvili et al., 2012a); besides, they have a stronger antimicrobial activity. This condition can provide advantages such as reduced toxicity, cost reduction, and overcoming resistance compared to conventional antibiotics. Components such as protein, polysaccharide, vitamins, or alkaloids in the structure of the biological material allow the production of nanoparticles by a biodegradable and nontoxic method by stimulating the formation of nanoparticles and preventing clustering during synthesis. However, the size, shape, or dispersion characteristics of the produced materials vary according to the used green approach. Thus, the biological activity of the produced NPs in parallel with the physical and chemical properties can also differ significantly. For this reason, it is highly important the selection of the green material to be used in nanomaterial production and in the screening methods to be used to determine the effectiveness of the product.

Algae are an important phytochemical source for metal nanoparticle synthesis and are naturally available (Rajeshkumar et al., 2013). The high metal uptake potentials of algae make them a low-cost material, and, since they are a renewable resource, they provide highly effective results in NP production through green synthesis. 

Gold is an important material for nanotechnological applications due to its resistance to surface oxidation and chemical inertness. AuNPs do not have toxic effects on human cells and are used in deep tissue imaging (Vigneron and Caps, 2016).

There are many studies in the literature on intracellular and extracellular synthesis of different metal nanoparticles and their antimicrobial potentials (Konishi et al., 2006; Khan et al., 2019; Wani and Ahmad, 2013; Shankar et al., 2016; Arsiya et al., 2017). In recent years, algae or blue-green algae-assisted synthesis has attracted considerable attention for the use of AuNPs in various applications (Kalabegishvili et al., 2012b; Annamalai and Nallamuthu, 2015). The presence of carotenoids, polysaccharides, proteins, and phenolic compounds in *Chlorella *green algae is important for pharmaceutical applications. *Chlorella* green microalgae can also serve as an effective metal reducing agent and capping agent with its components and has a strong potential for green synthesis (Annamalai and Nallamuthu, 2015). In addition, the carboxyl, hydroxyl, and amine functional groups in their structure are used to stabilize the NPs. The most studied species of this genus in the literature is *C. vulgaris,* but *C. sorokiniana* species was selected for this study because of its fast growth rate, high light tolerance, and strong accumulation capacity (Spencer-Milnes, 2019). To the best of our knowledge and understanding, this study is the first report of the production of AuNPs by green synthesis using local *C. sorokiniana* green algae. With detailed optimization studies, it is aimed to provide important information about the stabilization of AuNPs and to produce stable, small-sized, and effective nanoparticles with a local algae species obtained from our country’s resources. In addition, there is no study comparing the ultrastructural effects of the obtained gold nanoparticles on important fungal pathogens including *C. tropicalis*, *C. glabrata,* and *C. albicans* isolates with TEM analysis.

## 2. Experimental methods

### 2.1. Microorganisms

In our study, two clinical and one reference *Candida* isolates (*C. tropicalis *1660, *C. glabrata* 1744 and *C. albicans* ATCC 14053) obtained from Eskişehir Osmangazi University, Faculty of Medicine, Department of Microbiology. For identification of these isolates, germ tube test, microscopic morphology examination in Cornmeal Tween 80 agar, carbohydrate fermentation tests, and API 20C (bioMerieux, Marcy I’Etoile-France) commercial assimilation test were used. Yeast extract peptone dextrose (YPD) with glycerol (20%) was used to store the isolates and was stored at –80 ° C. Isolates removed from stock for fresh culture were incubated in RPMI 1640 medium at 37 °C for 24 h (CLSI M27-A2).

### 2.2. Collection and preparation of algae

In our study, stone, mud, and plant samples from State Hydraulic Works (DSİ, Turkey) watering pond (Musaözü) (39°41′51″ North, 30°19′25″ East) on Eskişehir-Kütahya road, 21 km from the center, were collected in glass bottles filled with lake water. Materials taken by scraping technique from these samples brought to the laboratory environment were transferred to solid medium containing BG-11 with 2% agar by smear technique. These petri dishes were kept at 28 °C under 3000 lux light for 12 h night and 12 h day. Colonies with different colors were detected. These colonies were transferred to liquid medium containing BG-11. Dilution method was applied to obtain single-cell culture (Andersen and Kawachi, 2005). In this method, mixed cultures transferred to the liquid medium were placed in the petri dish and diluted with sterile water. This dilution procedure was repeated several times until a single cell was found in the suspension. This cell was then isolated under an inverted microscope with the help of a Pasteur pipette. Pasteur pipettes were thinned under the fire as much as possible before using the tweezer. With these thin tips, a single cell was isolated and transferred to the liquid medium. These proliferating cells were diagnosed according to their morphological characteristics (Wehr et al., 2003; John et al., 2003; Tsarenko et al., 2006). According to these results, *C. sorokiniana *freshwater green algae culture was kept at 26 ± 2 °C in 100 rpm in a thermostatically controlled shaking incubator and illuminated with 3000 lux fluorescent lamps in a 12: 12 h bright dark environment. The growing algae cells were collected when they switched to the log phase.

### 2.3. Intracellular synthesis

*C. sorokiniana* freshwater green algae culture was kept at 26 ± 2 °C in a thermostatically controlled shaking incubator at 100 rpm and illuminated with fluorescent lamps at a density of 3000 lux in a 12: 12 h bright environment. The growing algae cells were collected when they switched to the log phase. For this process, it was washed with double distilled water for 10 min at 4500 rpm. This process was repeated 3 times. The collected cell mass (5 g) was resuspended in 100 mL of 1 mM HAuCl4 solution. A shaking incubator was in use at 26 ± 2 °C (100 rpm) for 48 h. In this process, the color change in the solution indicates the formation of AuNPs. In addition, bioreduction by *C. sorokiniana* cells was routinely monitored by measuring UV-Vis spectra (Focsan et al., 2011; Parial et al., 2012; Suganya et al., 2015; Kumar et al., 2016).

### 2.4. Preparation of algae extracts and extracellular synthesis 

*C. sorokiniana* isolates were centrifuged at 4500 rpm for 10 min and washed once with ultrapure water. It was heated at 80 °C for 20 min to obtain algae extract and filtered using Whatman No. 1 filter paper. They were stored in the refrigerator until use. This obtained aqueous extract was centrifuged (20 °C, 4500 rpm, 10 min) to avoid leaving cell residue. The synthesis process was implemented by modifying the methodology of Swain et al. (Swain et al., 2016). In particular, optimization conditions were tried and revised. 1 mL of *C. sorokiniana* extract obtained was mixed using 2.5 mL of heated magnetic stirrer from 1 mM HAuCl4. During the reaction period, the color changed from light green to pink-purple color. This color change in colloidal solutions indicates the formation of AuNPs. AuNPs obtained after extracellular synthesis were stored at +4 °C and in the dark because higher temperatures may cause aggregation problems and storage in the dark minimizes photoinduced oxidation (Zayadi et al., 2020).

#### 2.4.1. pH optimization in AuNPs synthesis 

The pHs of the solutions prepared with 1 mL *C. sorokiniana* extract and 2.5 mL (1 mM) HAuCl4 were prepared to have pH value of 4, 5, 6, 7, 8, 9, respectively, using HCl / KOH (80 °C). Then, pH value optimization was made according to UV-Vis measurements.

#### 2.4.2. Metal salt optimization in AuNPs synthesis

After 1 mL of *C. sorokiniana* extract and 2.5 mL of HAuCl4 solution were prepared at 0.5 mM, 1 mM, and 5 mM molarities, optimum pH value and 80 °C, UV-Vis measurements were taken, and the metal salt optimization was performed.

#### 2.4.3. Time optimization in gold nanoparticle synthesis

1 mL of *C. sorokiniana *extract and 2.5 mL of HAuCl4 (1 mM) solution were prepared at the optimum pH value and 80 °C, and UV-Vis measurements were taken in 1 th, 3 th, 5 th, 10th, 15th, 30th, 45th, 60th, 75th, 90th min and 24th h. Thus, time-optimization was performed.

### 2.5. Characterization of AuNPs

#### 2.5.1. UV-Vis

UV-Vis spectra were recorded with UV-Vis (AE-S90-2D Spectrophotometer, Guangdong, China) device at room temperature. Measurements were recorded at the maximum absorbance wavelength to determine the spectra of our optimization reactions (pH value, salt, and time). All scans were done at a wavelength of 190–1100 nm.

#### 2.5.2. Particle size and Zeta potential measurements 

The optical properties of nanoparticles were examined by spectral analysis. The absorbance spectrum of nanoparticles was determined using a spectrophotometer and a 10 mm path length quartz cuvette. Particle size, zeta potential, electrical conductivity measurements of nanoparticles synthesized by green synthesis were made using zetapotantiometer (Malvern–Zetasizer (Nano-Z590, Cambridge, United Kingdom). Three measurements were made consecutively, and the temperature of the device was adjusted as 25 °C and the light scattering angle as 90° during the measurements (Malvern CGS-3).

#### 2.5.3. FE-SEM and TEM

In our study, the electron microscope was used in two stages: In order to determine the surface characteristics with FE-SEM analysis, samples were dropped on Whatman No. 1 paper and placed on stubs attached with carbon tape after drying. Then, they were examined with field emission scanning electron microscope (FE-SEM). In order to determine the morphological properties of the samples by transmission electron microscopy (TEM), they were dropped on copper grids from the same samples and examined with TEM after they were dried thoroughly (Hitachi HT 7800), and elemental analysis was performed with the energy distribution X-Ray spectroscopy (TEM-EDX) detector.

#### 2.5.4. Fourier Transform Infrared Spectroscopy (FTIR) 

Prior to the analysis, samples were washed three times with distilled water to remove organic compounds that were not bound to the nanoparticle surface. Then, nanoparticles were lyophilized and solidified, and potassium bromide (KBr) was heated for 1 h at 100 oC, and after removing moisture, it was converted into a fine powder in a mortar of approximately 3 mg KBr and put into the press machine, and a disc was obtained as a thin film in 3000 bar pressure. This disc was placed in the FTIR device in a spectra ranging from 400–4000 cm–1 were taken. Thus, the surface chemistry of the reduced gold nanoparticles and the analysis were performed to check the presence of the biofunctional portions of the extracts. 

#### 2.5.5. X-Ray diffractometer (XRD)

Panalytical Empyrean X-Ray diffractometer (XRD) was used to determine the crystal structure of the nanoparticles, and the powder diffraction pattern analyzes of the samples were taken. In the XRD device, using the Cu K tara tube (λ: 1.54 Å), 2θ angle scanning was performed under 45 kV voltage and 40 mA current analysis conditions. With this method, the structure that is in pure crystal form is resolved, chemical analysis of the bond length, bond angles and three-dimensional structure of the unit cell could be provided. 

#### 2.5.6. Inductively coupled plasma-mass-spectrometry (ICP-MS)

ICP-MS was used for the quantification of synthesized nanoparticles (Thermo iCAP RQ ICP-MS). This process was accomplished by ionizing the biosynthesized C-AuNPs in solution with inductively coupled plasma (Ar plasma) and then determining the mass/charge (m / z) ratios using a mass spectrometer to separate and measure these ions. Before the analysis, the sample was dissolved in the microwave, and its organic content was burned using acid in different proportions (HNO3 and H2O2). After removing the organic content, dilutions were performed, and a calibration curve was created from the stock standard solution (Redox-423A) during analysis. Quantification was made according to the dilution factor and standard curve. The results obtained here were used specifically to determine antifungal activity and formed the initial concentration of test analyzes.

### 2.6. Evaluation of the antifungal effect of NPs

#### 2.6.1. Antifungal susceptibility tests

The antifungal susceptibilities of the synthesized nanoparticles were evaluated by agar diffusion and broth microdilution (minimum inhibitor concentration-MIC) tests. 

#### 2.6.2. Agar diffusion test 

Yeast extract peptone dextrose (YPD) agar medium was used in agar diffusion method to investigate the antifungal activity of AuNPs. Activated microorganisms were set to McFarland 0.5, and they were inoculated to plates. AuNPs impregnated to discs at 10 µL were placed in the medium. The formed zones after 24 h were measured and the image was taken with stereo microscope (ZEISS Stemi 508). The experiments were repeated 3 times (Jorgensen et al., 2015).

#### 2.6.3. Minimum inhibitor concentration (MIC) and minimum fungicidal concentration (MFC) tests

Sterile, U-based and 96-well microplates were used for antifungal susceptibility testing. After the serial 2-fold dilutions of the active ingredients were prepared, the density of the test organisms in the RPMI 1640 medium in the well was adjusted to be 0.5–2.5×103 CFU/mL. After a 48 h incubation at 37 °C, absorbance measurements were made at 600 nm with the aid of a microplate reader. The lowest nanoparticle concentration that inhibits microbial growth was determined as the MIC value. 

To determine the minimum fungicidal concentration (MFC) value, 50 µL of clean wells containing concentration below the MIC value was taken and inoculated in YPD plates and incubated at 37 °C for 48 h. The lowest concentration that does not show *Candida* growth on the agar surface was determined as MFC.

#### 2.6.4. Investigation of the ultrastructural effect of AuNPs on Candida isolates by transmission electron microscopy (TEM) 

Cells were adjusted to 105 CFU / mL, washed 3 times with 1×PBS (phosphate-buffered saline), and then treated with the active substance at room temperature and untreated cells were taken in 2.5% glutaraldehyde prepared in PBS and fixed for 24 h. Samples washed 3 times with PBS were taken for 1 h secondary fixation in 1% osmium tetraoxide. Samples washed again with PBS buffer were passed through the alcohol series for dehydration processes (10 min in 30% ethanol + 10 min in 50% ethanol + 10 min in 70% ethanol + 10 min in 90% ethanol + 10 min in 95% ethanol + 10 min in 100% ethanol). Subsequently, samples were taken to propylene oxide for 10 min at room temperature for clarification and then kept in rotator for 10 min in propylene + araldehyde mixture. Finally, samples were taken into pure araldite and left on rotator for 1 day. Cells taken into araldite-based embedding material were allowed to polymerize and block for 48 h at 60 °C. The ultrathin sections (60 nm) of the samples taken with ultramicrotome (Leica Ultracut R, Leica Microsystems GmbH, Wetzlar, Germany) were also examined on Hitachi HT 7800 TEM after being taken on the grid with uranyl acetate-lead citrate (Børsheim et al., 1990; Öztürk et al., 2020). 

## 3. Results

Our test isolate *C. sorokiniana* is a single-celled green microalgae belonging to the Chlorophyta division. It has a spherical shape and is approximately 2 to 10 μm in diameter and not flagellated. The chloroplast of this algae is rich in green photosynthetic pigments, contains a lot of fat and is used to investigate the way to increase biofuel efficiency (Cazzaniga et al., 2014). In our present study, when exposed to gold ions compared to control biomass, algae biomass changed markedly from natural bright green to pinkish-purple compared to the control biomass. This suggested that Au^III^ ions are reduced to Au0.

**Figure 1:**
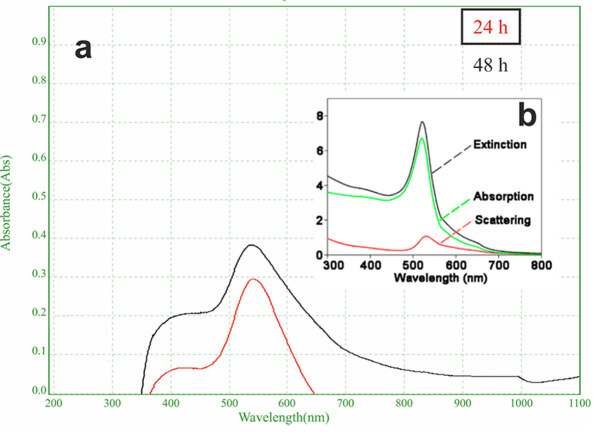
(a) UV–visible absorption spectrum of AuNPs of intracellularly synthesized using C. sorokiniana after 24 h and 48 h reaction (b) The standard spectrum of AuNPs was calculated based on the study of Yang et al., 2015.

The result of UV-Vis spectral analysis which is carried out at 24th and 48th h after exposure of algal biomass supports this situation (Figure 1). For further verification, a SPR band was formed after the first 24 h later and absorbance value was measured at 547 nm. After 48 h, it was determined that the spectral peak expanded and the absorbance value was 554 nm. These results showed that the nanoparticles formed are intracellular. In order to image the intracellular accumulation, ultrathin sections of control sample and algae biomass exposed to HauCl4 were taken and examined by TEM (Hitachi HT 7800).

**Figure 2:**
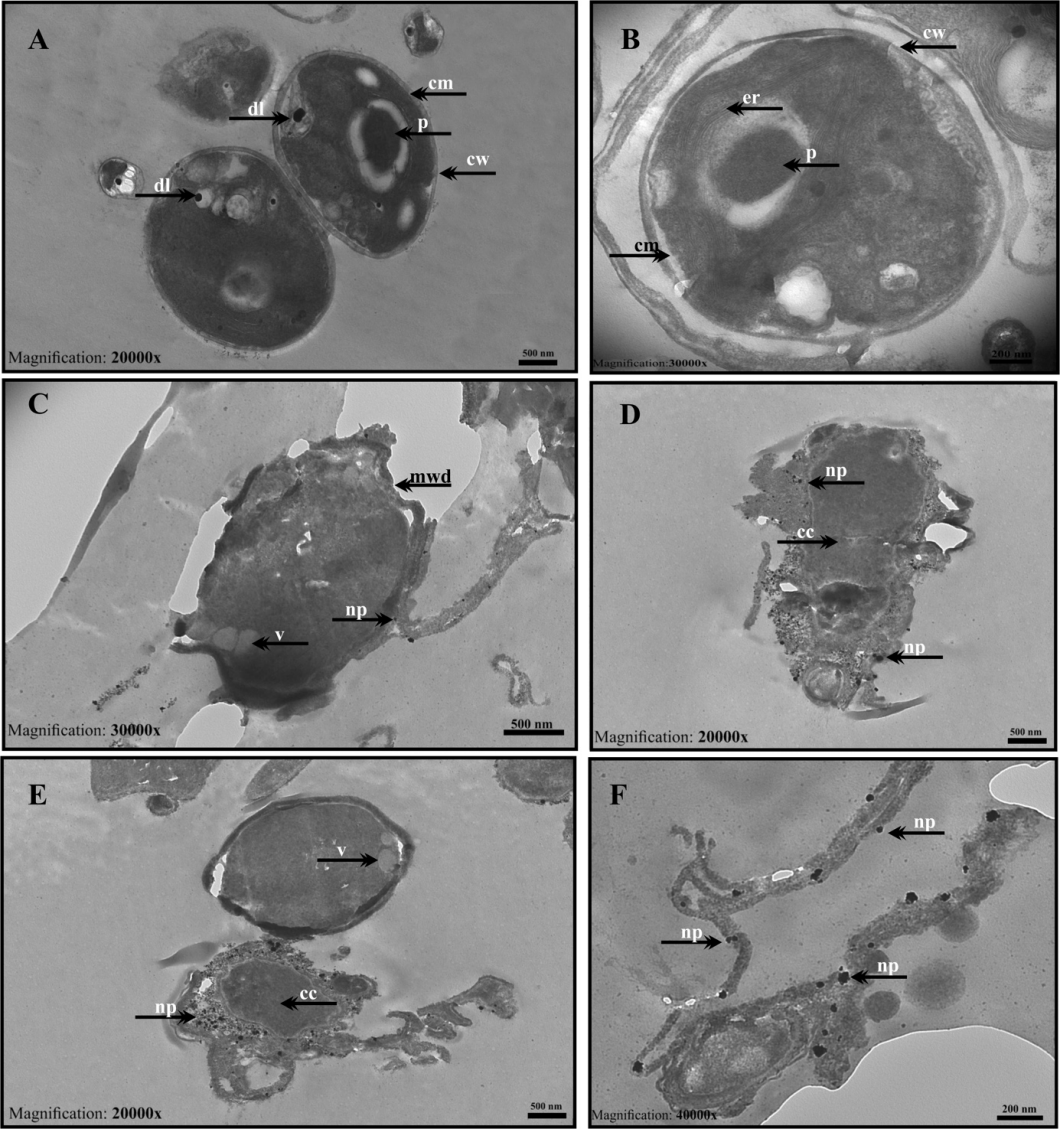
TEM micrographs of intracellular synthesis of AuNPs using C. sorokiniana (a, b) Control Chlorella cells cultured with BG-11 medium only showing regular cellular morphology: cell wall (cw), cell membrane (cm), lipid droplet (ld), thylakoids (thy) and pyrenoid (p); (c, d, e, f) Cells treated with 1 mM Au solution for 24–48 h indicate ultrastructural changes in the cell structure: membrane wall damage (mwd), nanoparticles on cell wall (np), vacuoles (V), and cytoplasm condensation (cc) (Scale bar: 500 nm for a-c-d-e images; 200 nm for b and f images).

TEM micrographs showing the intracellular biosynthesis of AuNPs using *C. sorokiniana* is presented in Figure 2. Untreated control *C. sorokiniana* cells were cultured with BG-11 medium only. Cells have a regular morphology; whole-cell wall and membrane structure were observed (Figure 2a, 2b). After the treatment of AuNPs, significant damage was observed on the cell surface and NPs accumulated on the cell surface. Membrane and wall damages, vacuole formations, and cytoplasm condensation (cc)’s were detected (Figure 2c, 2d, 2e, 2f). When we evaluate the synthesized nanoparticles in size, it is seen that they are 20–40 nm and spherical shaped (Figure 2 f). TEM findings showed the cells were damaged after treatment with 1 mM HauCl4 in intracellular synthesis; thus, the continuation of the study was continued with extracellular biosynthesis.

**Figure 3:**
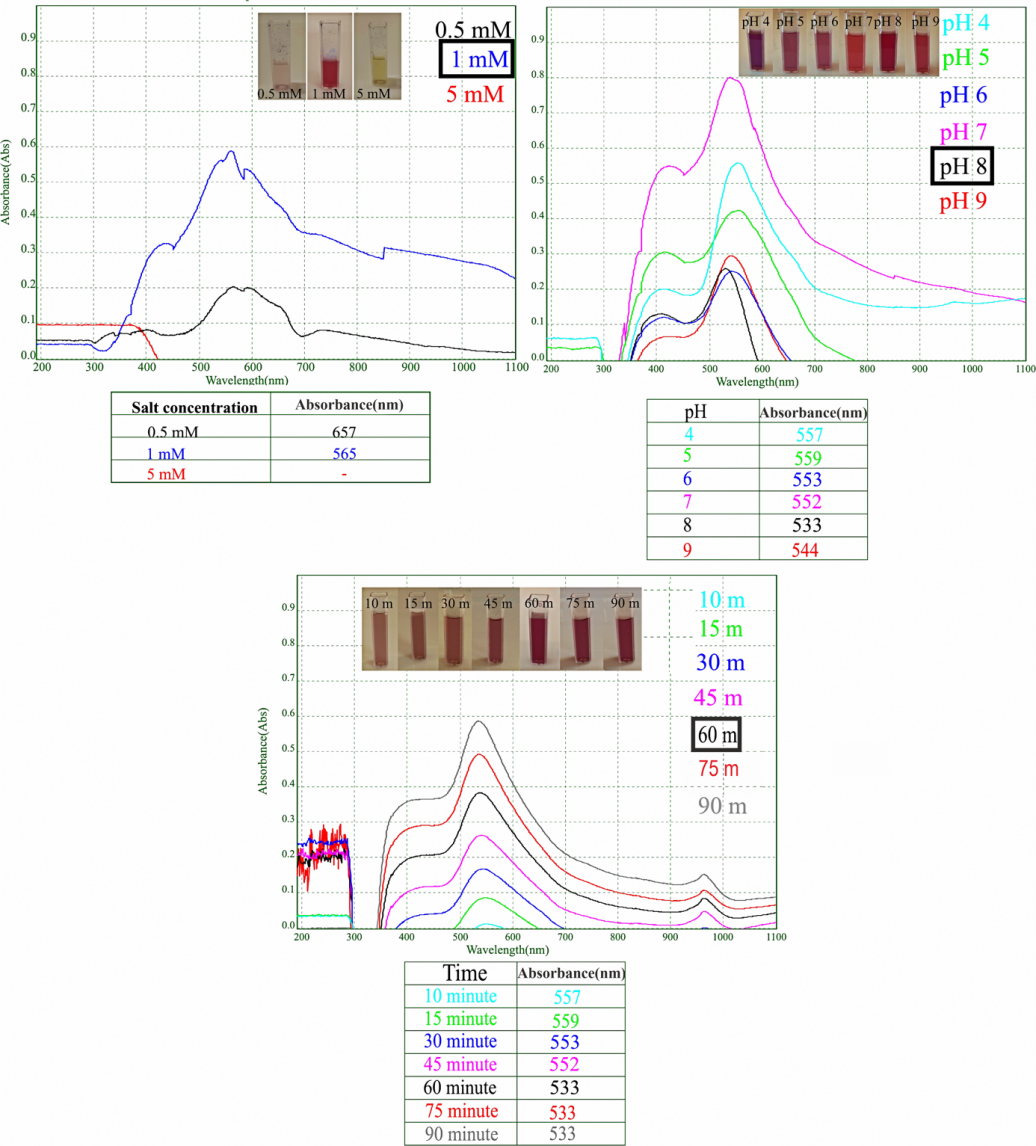
UV-visible absorbtion spectra of extracellulary synthesized C-AuNPs recorded for optimization of (a) salt concentration, (b) pH, (c) time parameters.

In our extracellular biosynthesis study, experimental conditions such as pH value, time, and salt were optimized for gold nanoparticle synthesis. The appearance of the characteristic surface plasmon resonance (SPR) band of AuNPs confirms nanoparticle formation. This band can be understood by spectra that nanoparticles in the reaction medium can form in various sizes and shapes. In particular, less dense and broad spectra gave an idea about these nanoparticles. In line with these results, optimum gold nanoparticles were synthesized. The synthesis of AuNPs was monitored by color change and UV vis spectroscopy. The formation of gold nanoparticles with receiving pink-reddish color appearance of the solution was confirmed (Figure 3).

### 3.1. Optimization of extracellular biosynthesis 

#### 3.1.1.Salt optimization studies

Three different concentrations were studied for salt optimization. When 0.5 mM HAuCl4 was used as precursor, absorbance was determined at 657 nm, and nanoparticle synthesis was not at the desired level. When 5 mM HAuCl4 was used, nanoparticle formation was not observed. Using 1 mM HAuCl4 showed that small and dispersed gold nanoparticles were synthesized by giving absorbance at 565 nm. By considering these results, the optimum HAuCl4 concentration for the AuNPs synthesis was determined as 1 mM (Figure 3a). 

#### 3.1.2. pH value optimization studies

In our study, the effect on AuNPs production of different pH values was investigated by altering the pH value of the synthesis environment between 4 and 9, results of which are presented in Fig 3b**.** According to the reaction color and the intensity of the SPR bands, pH = 8 was determined as the optimum value. For this reason, pH value of 8 was used for the next studies (Figure 3b).

#### 3.1.3.Time optimization studies

The reduction of Au3+ ions to Au0 mediated by *C. sorokiniana* extract was analysed spectroscopically at different reaction time. The synthesis of AuNPs started within 10 min, which showed an SPR band at 557 nm. As the reaction continued, the density of the SPR band changed towards 533 nm, indicating that spherical nanoparticles were formed. The synthesis was completed within 60th min, and no further rise in the SPR band intensity was detected. (Figure 3c).

### 3.2. Characterization of AuNPs 

**Figure 4:**
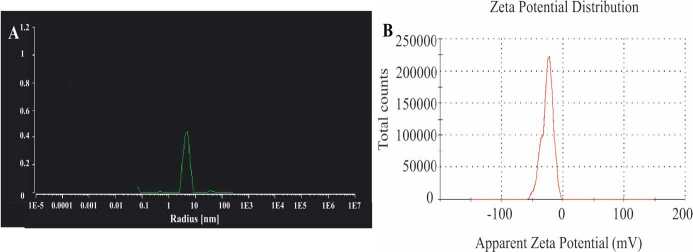
DLS and Zeta potential diagrams of C-AuNPs (a) To determine size distribution of C-AuNPs (DLS) (b) Zeta potential to determine the stability of C-AuNPs.

C-AuNPs synthesized as green synthesis and determined by DLS study are shown in Figure 4a. As shown in the DLS plot, it was observed to produce a uniform particle size, and it was found to support the dimensions obtained in TEM (5–15). In addition, if the PDI (polydispersity index) value is between 0.1–0.25, narrow distribution is obtained close to the desired monodisperse, while it is above 0.5 means wide distribution due to large particles and aggregate formation (Nidhin et al., 2008; Tripathi et al., 2010; Kavaz, 2011; Mohammadpour et al., 2012). As a result of our study, the PDI value of C-AuNPs is between 0.38, that is, between narrow distribution and wide distribution. Zeta potential was used to determine the stability, total charge, and surface charge of the synthesized AuNPs. Potential Z value of –28.8 mV was obtained, indicating that biosynthesized nanoparticles as C-AuNP formed a stable colloidal suspension with negatively charged particles (Figure 4b).

#### 3.2.1. FE-SEM analysis

**Figure 5:**
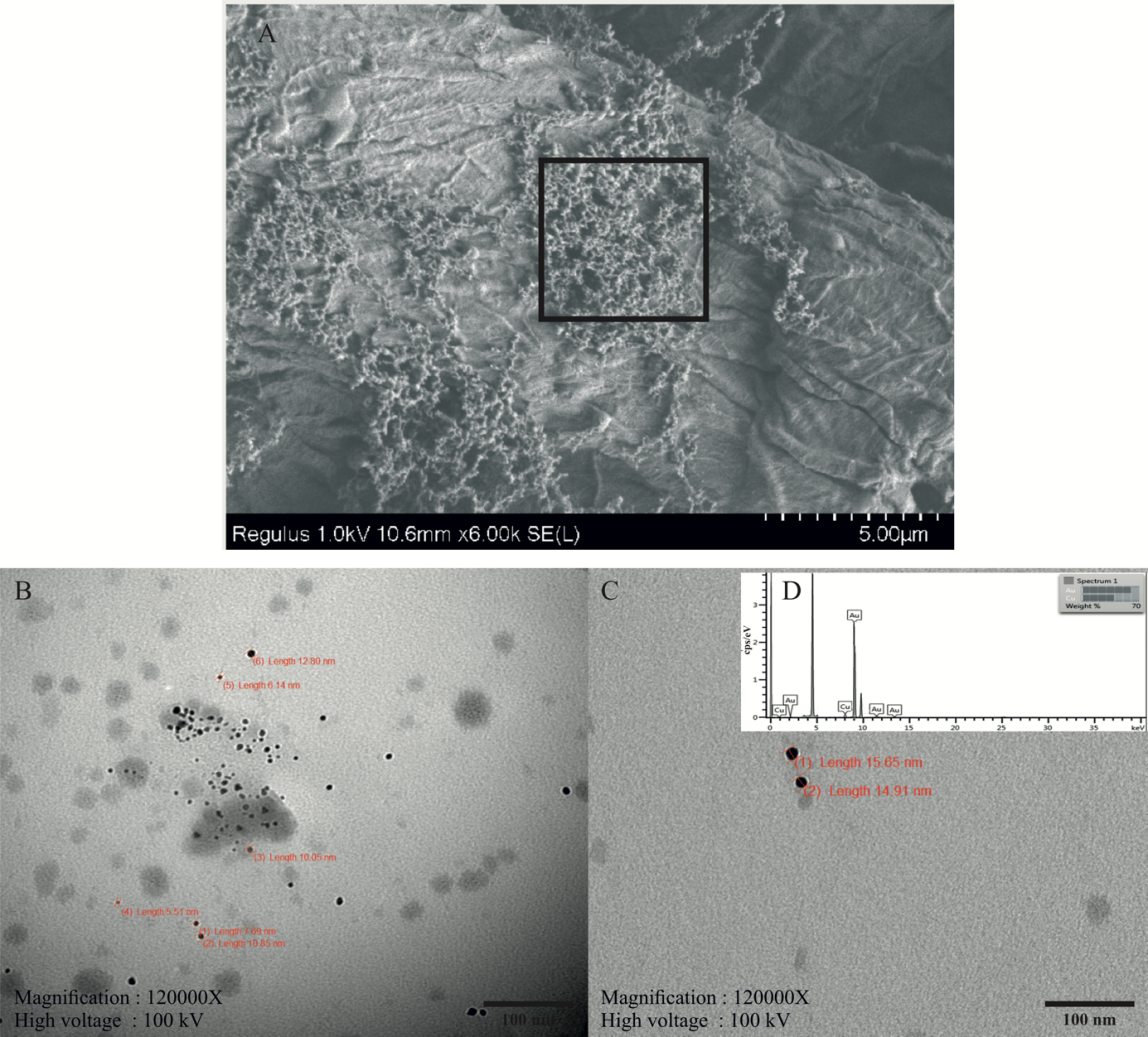
SEM and TEM images of C-AuNPs (a) SEM images and morphology of synthesized C-AuNPs. (b, c) TEM images and size values of synthesized C-AuNPs at (100 nm scale); (d) Analysis of energy dispersive X ray (EDX) spectrometer of AuNPs.

FE-SEM was used to image and morphologically characterize AuNPs. Figure 5a shows the nanoparticles that spread spherically.

#### 3.2.2. TEM analyses

HR-TEM was used to determine detailed information about the size, morphology, and elemental analysis of AuNPs. The AuNPs from C-AuNP were spherically shaped, had a particle size of 5-15 nm on average, and homogeneously dispersed. In addition, it was observed that it formed in triangular shapes in some places (Figure 5b, 5c). The composition and purity of nanoparticles synthesized with the TEM-EDX detector (Oxford Instruments X-MaxN) have been verified and the EDX spectrum showed elemental signals of Au atoms (Figure 5d).

#### 3.2.3. Fourier-transform infrared spectroscopy (FTIR) analysis

**Figure 6:**
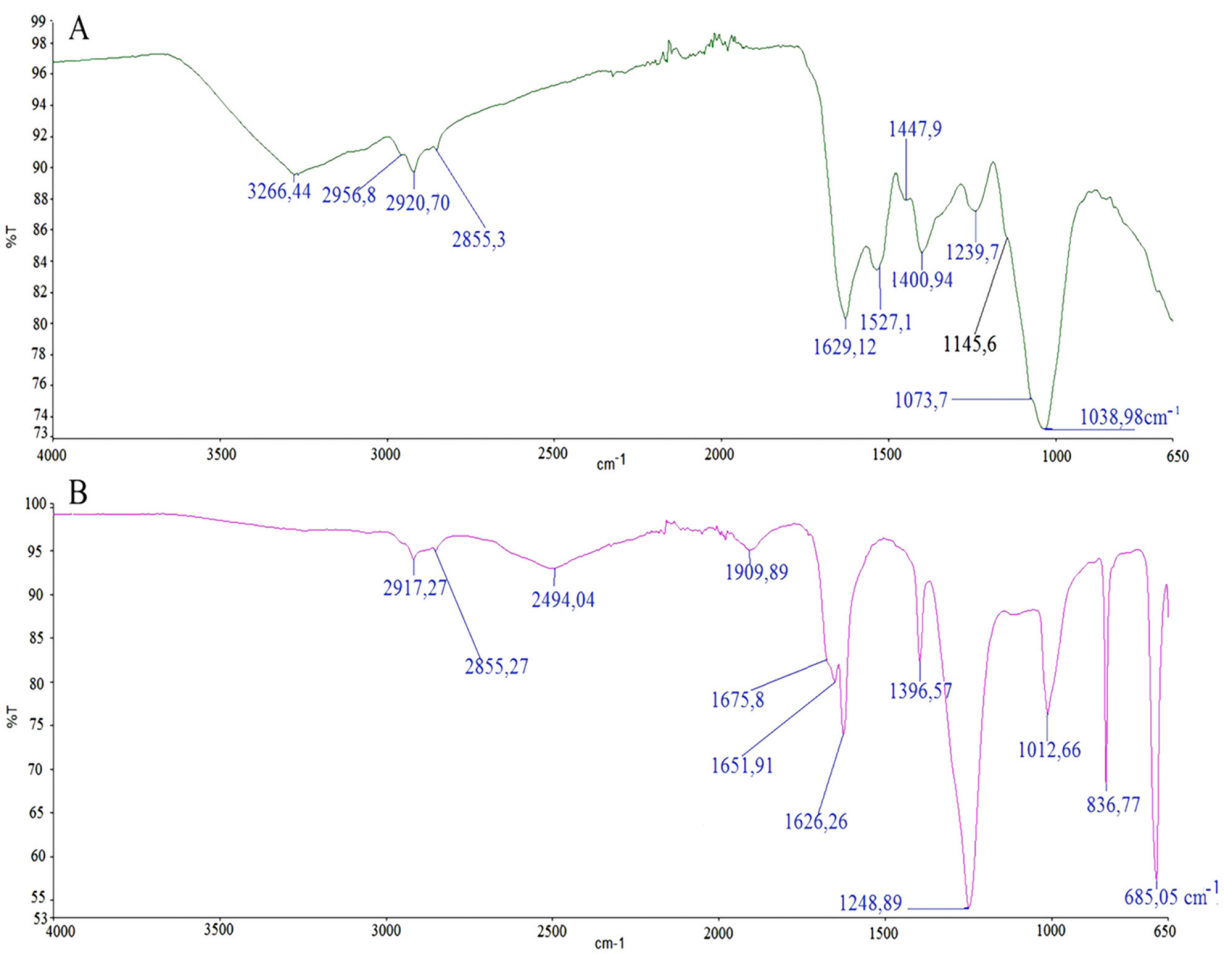
FTIR analysis. FTIR spectra of C. sorokiana (a). Synthesized AuNPs (b).

FTIR analysis was carried out to identify the functional groups involved in metal reduction when synthesizing AuNPs. According to the spectrum measurements, for *C. sorokiniana* extract, bands were seen at 3266.44, 2956.8, 2920.70, 2855.3, 1629.12, 1527.1, 1447.9, 1400.94, 1239.7, 1145.6, 1073.7 and 1038.95cm–1, respectively. For C-AuNP, bands were seen at 2917.27, 2855.3, 2494.04, 1909.89, 1675.8, 1651.91, 1626.26, 1396.57, 1248.89, 1012.66, 835.77, and 685.05cm–1, respectively (Figure 6). The FTIR spectrum at 3266.44 cm–1 represents the location of the N-H band and the weak carbonyl band. It is seen that this spectrum has completely shifted during the gold reduction. After reduction, large dimer bands emerged as a result of O-H stretching, and these bands are formed by matching the C-O stretching of the dimer with the O-H in-plane bending.

C = C stretching for FTIR spectra presence of alkenes (1629.12); N-H bending, and N = O stretching have the presence of carbonyl compounds (1527.1); N-O stretching exists in the presence of peptides (1447.9); SO2 asymmetric stretching showed the presence of proteins and glucosinates (1400.94). It was observed that these bands disappeared completely during the gold reduction. These functional groups are thought to be active bonds in reduction.

The aromatic C-H in-plane bending disappeared at 1073.7 and 1038.95 cm–1 and shifted towards the 835.77 cm–1 band. This suggests that aromatic structures are effective in the reduction of gold ions. It is predicted that algae pigments such as carotenoid may have been involved in gold reduction (Figure 6 a, b).

#### 3.2.4. X-ray diffraction (XRD) analysis

**Figure 7:**
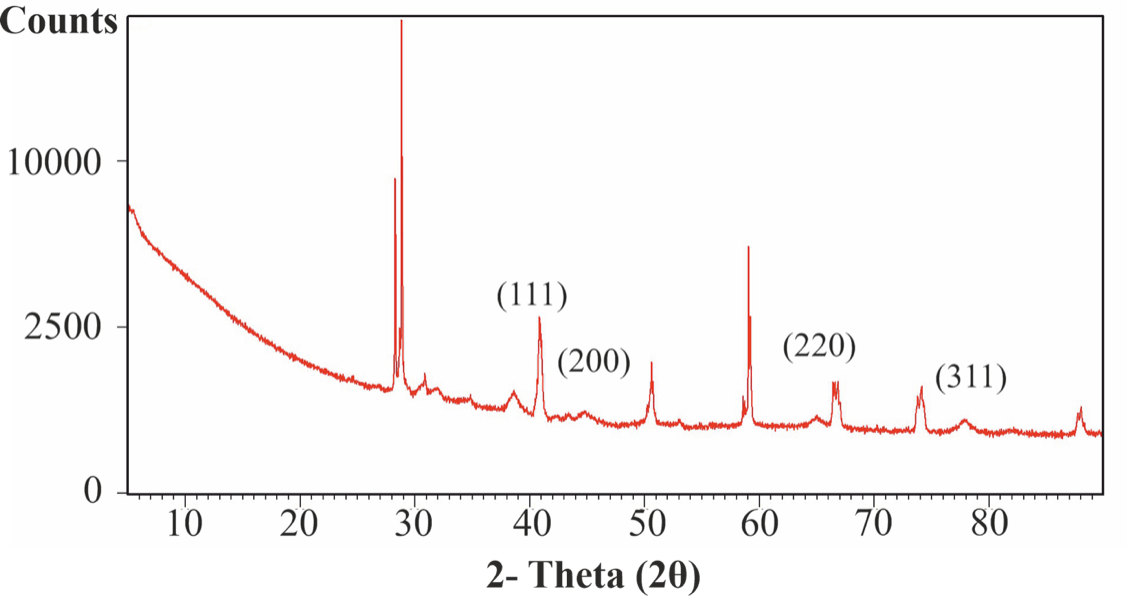
XRD pattern analysis of synthesized AuNPs using C. sorokiana.

X-ray diffraction studies have been performed to confirm the crystalline nature of AuNPs. The reported XRD pattern is reflections (111), (200), (220) and (311), respectively. In our results, these reflections were seen as 2λ = 38.1°, 45.1°, 64.6° and 77.4° diffraction peaks (file no: 01-071-4614). The presence of dense peaks corresponding to nanoparticles matched with the Au reflections defined by Bragg’s diffraction pattern. According to this pattern, a strong diffraction peak located at 38.1° on the surfaces of the face-centered cubic metal gold structures (111) was seen stronger than the refractive peaks of the other three faces. (Figure 7). In addition to the characteristic peaks, peaks of different sizes are also noticed. This is thought to be due to the extract used during the synthesis of the nanoparticle. Our XRD results showed that the nanoparticles obtained by the green synthesis method have a crystal structure (Figure 7).

### 3.3. Antifungal activity 

**Figure 8:**
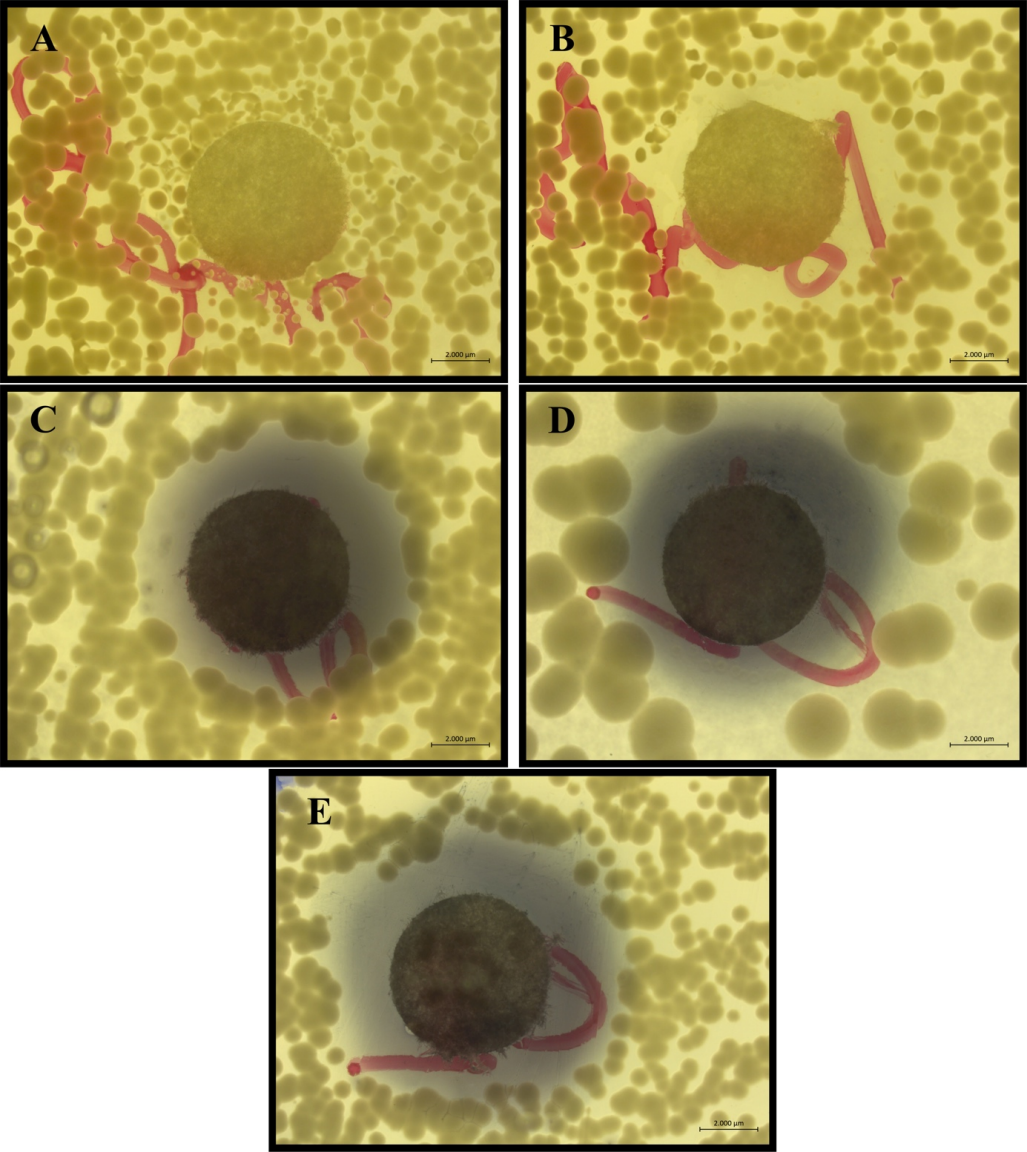
Antifungal activity of C-AuNPs on Candida isolates by disc diffusion (a). C. sorokiana extract. (b). Amphotericin B, an antifungal standard. (c). C. albicans ATCC 14053 treated with C-AuNPs. (d). C. tropicalis 1660 treated with C-AuNPs. (e). C. glabrata 1744 treated with C-AuNPs.

The antifungal effects of AuNPs were tested on *C. albicans*, *C. tropicalis,* and *C. glabrata* yeast isolates by agar diffusion test and the results are presented in Figure 8. Amphotericin B was used as a control drug. AuNPs showed a larger zone diameter than Amphotericin B on *Candida* isolates and were found to be more effective.

Our broth microdilution MIC results reveal that AuNPs show the same MIC values in all isolates (Table ). According to the results of ICP-MS analysis, the concentration range of AuNPs was measured as 600 μg/mL. *C. albicans* ATCC 14053 isolate with the MIC of 4.31 μg/mL showed the most effective result for AuNPs. In addition, when the MFC values of the isolates are taken into consideration, a two-fold increase has been determined compared to the MIC values. The antifungal effect of gold nanoparticles was found to be higher on *C. glabrata* 1744 isolate compared to *C. tropicalis* 1660 isolate.

**Table:** Antifungal activity of C-AuNPs on Candida isolates by disc diffusion and broth microdilution tests: MIC (µg/mL), MFC (µg/mL) and the diameters of zone inhibition.

Fungal Pathogens	Amphoterisin B			C. sorokiniana extract			Biosynthesized Gold Nanoparticle		
	Disc diffusion assay (mm dia)	MICµg mL-1	MFC µg mL-1	Disc Diffusion Assay (mm dia)	MIC µg mL-1	MFC µg mL-1	Disc Diffusion Assay (mm dia)	MIC µg mL-1	MFCµg mL-1
C. albicans	8 ± 0.2	3.13 ± 0.8	6.25 ± 1.2	-	-	-	10 ± 0.3	4.31 ± 1.3	8.6 ± 0.8
C. tropicalis	8 ± 0.2	6.25 ± 0.7	12.5 ± 0.9	-	-	-	11 ± 0.2	17.2 ± 1.2	34.5 ± 0.8
C. glabrata	7 ± 0.1	3.13 ± 0.8	6.25 ± 1.0	-	-	-	11 ± 0.4	8.6 ± 0.9	17.2 ± 1.2

#### 3.3.1. The ultrastructural effects of AuNPs on Candida isolates

**Figure 9:**
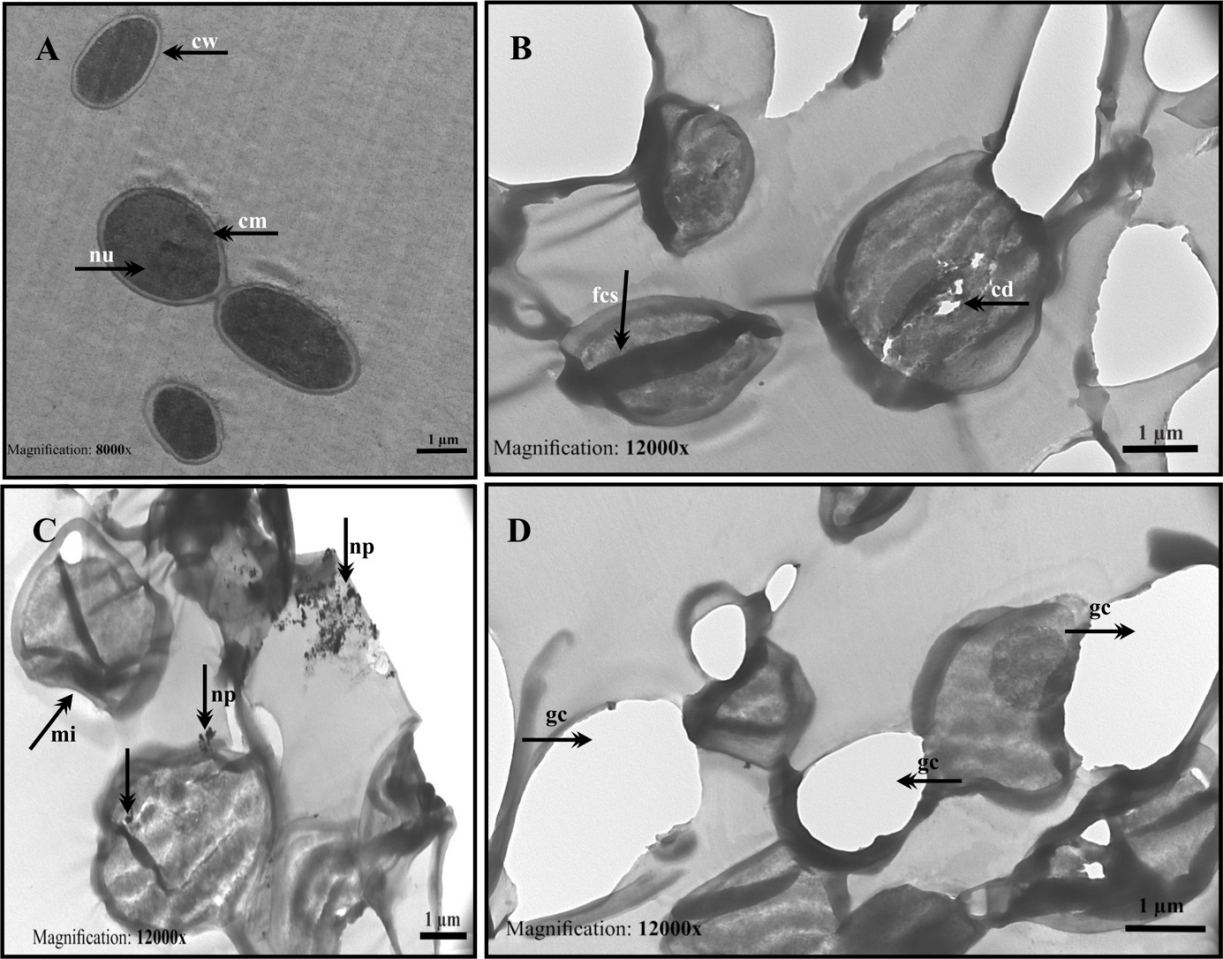
TEM micrographs of C. albicans ATCC 14053 treated with C-AuNPs. (a) Untreated control Candida cells; (b, c and d) Cells treated with C-AuNPs. Cells without treatment showed regular and well-conserved features, homogenous cytoplasm and distinctive membrane and wall structure: cell wall (cw), cell membrane (cm), nucleus (nu); After treatment with AuNPs, cytoplasm damage (cd), folded cell shapes (fcs), ghost cells (gc), nanoparticles (np), and membrane invagination (mi) were evident. (Scale bar: 1 μm for a-b-c-d images).

Ultrastructural changes of *C. albicans *ATCC 14053 isolate treated with green synthesized AuNPs were observed by TEM (Figure 9a). Untreated *C. albicans* cells were seen as regular oval and round; morphology, membrane, and wall structures show integrity, cytoplasm is homogeneous. However, damage findings were very high in cells treated with AuNP; cell deaths and nucleus irregularity have been detected (Figure 9b, 9c, 9d).

**Figure 10:**
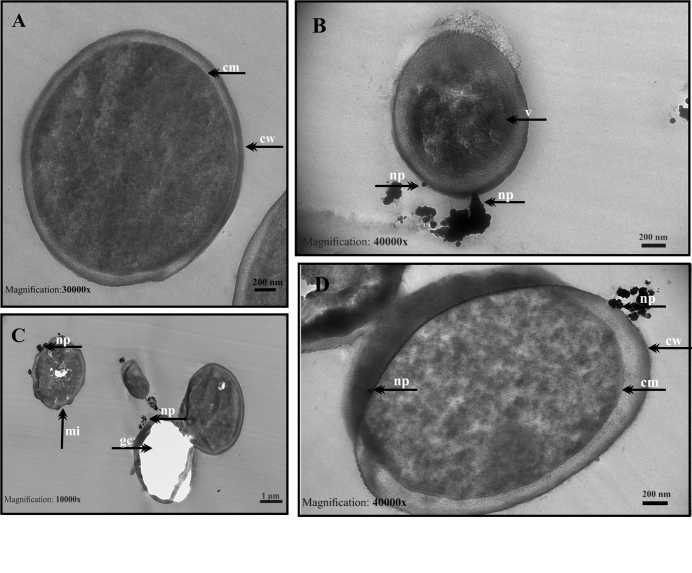
TEM micrographs of C. tropicalis 1660 treated with C-AuNPs. (a) Untreated control Candida cells; (b, c and d) Cells treated with C-AuNPs. Cells without treatment showed well preserved cellular morphology, and entire cell structure: cell wall (cw), cell membrane (cm); After treatment with C-AuNPs, ghost cells (gc), membrane invagination (mi), nanoparticles on the cell wall (np), and membrane ruptures (mr) were apparent. (Scale bars is 200 nm for a–b–d; 1 μm for c images).

Similarly, TEM images obtained from *C. tropicalis* 1660 isolate are presented in Figure 10. TEM images of control *C. tropicalis *1660 isolate showed well-preserved cellular morphology and entire cell structure (Figure 10 a). After the treatment with AuNP, ghost cells, folded cell shapes that have lost their regular structure after contact of the nanoparticles to the cell wall were observed (Figure 10b, 10c, 10d).

**Figure 11:**
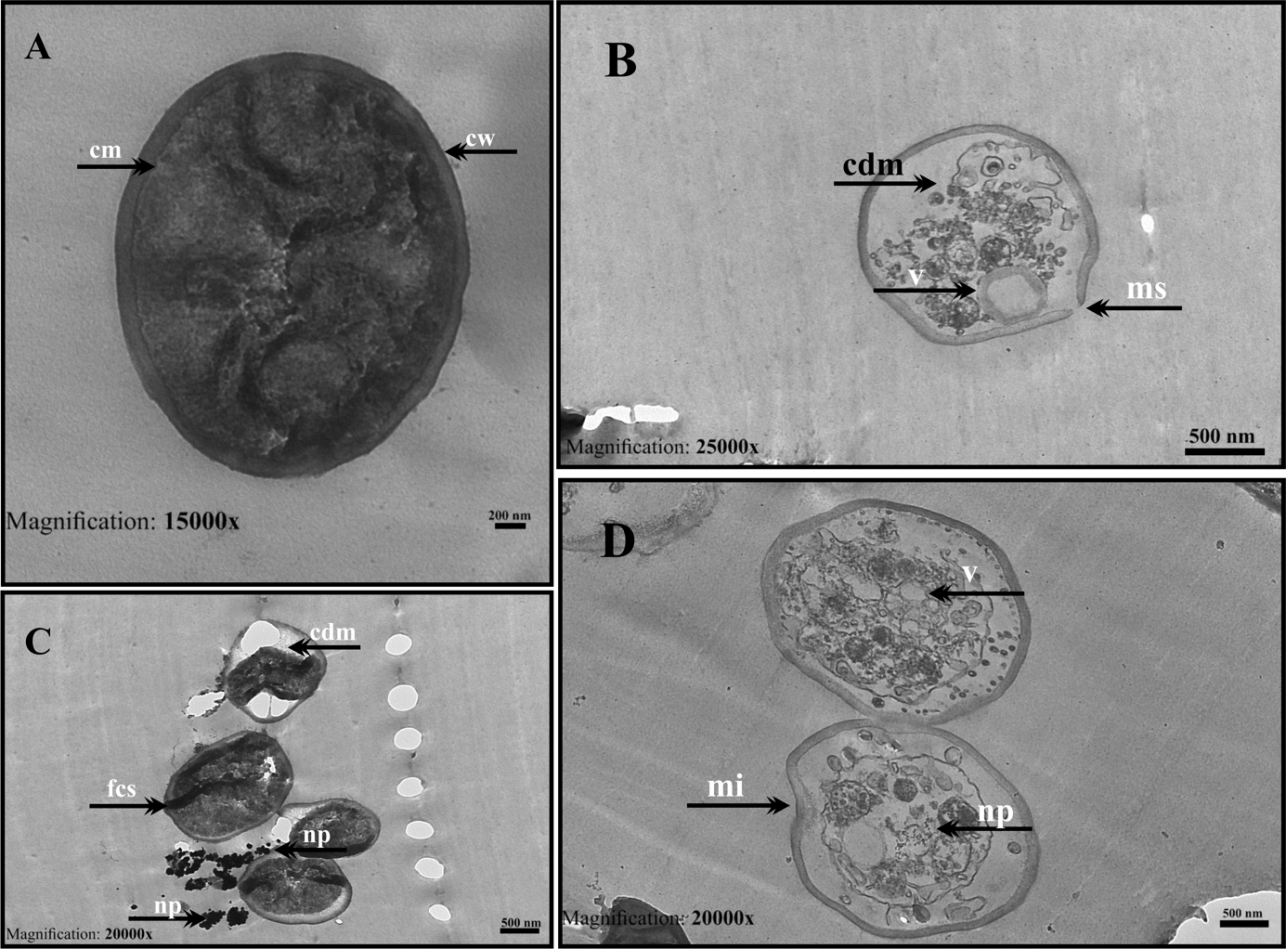
TEM micrographs of C. glabrata 1744 treated with C-AuNPs. (a) Untreated control Candida cells; (b, c and d) Cells treated with C-AuNPs. Control Candida cells show homogenous cytoplasm and regular morphological features: cell wall (cw), cell membrane (cm); After treatment with AuNPs, cytoplasm damage (cd), folded cell shapes (fcs), ghost cells (gc), nanoparticles (np), vacuoles (V), and membrane invagination (mi) were observed. (Scale bars is 200 nm for a image; 500 nm for b,c, and d images).

TEM images obtained from *C. glabrata *1744 isolate grown in the absence and presence with AuNP are presented in Figure 11. The control group cells show a homogeneous stoplasmic distribution and uniform shape with regular wall and membrane structure (Figure 11a). In AuNP-treated cells, cytoplasmic melts, organelle disruptions, cell membrane, and wall ruptures in some regions, lysed cells, cytoplasm withdrawal, abundant vacuol formation, vesicular structures, and intense involvement of the nanoparticles in the wall (Figure 11b, 11c, 11d).

## 4. Discussion 

Due to the spread of infectious diseases and an increase in drug resistance rates, new antimicrobials are being explored, and biosynthesized NPs are among the most promising therapeutic agents (El-Sheekh and El-Kassas, 2016). Among these biological systems, algae become prominent with many properties they possess. Algae are rich in lipids, minerals, vitamins, and some bioactive substances, and they have a high potential for use in medical and industrial fields. Since they contain hydroxyl, carboxyl and amino functional groups in their structure, they can perform metal nanoparticle synthesis in a single step. During the green synthesis, NPs can be synthesized in various sizes, shapes and morphologies, eco-friendly, safe and fast manner.

Herein, we firsly reported on the green synthesis of AuNPs from *C. sorokiniana* local algae. The synthesized nanoparticles are spherical shapes. According to the literature data, depending on the variety factors such as biological potential of the used algae and the synthesis method, the AuNPs shapes obtained can be spherical, triangular or rectangular (Chaudhary et al., 2020; Noruzi et al., 2011; Raghunandan et al., 2011). In a study of Parial et al., the properties of AuNPs obtained from different algae by green synthesis were compared, and it was stated that the size and shape of the NPs varied according to the type of microbial source (Parial et al., 2012). Similarly Menon et al. also explained that NPs are synthesized in different shapes depending on the used species (Menon et al., 2017). Therefore, AuNPs obtained with *Chlorella *are generally spherical in the literature and these data support our findings (Annamalai and Nallamuthu, 2015).

Venkatesan et al. synthesized AuNPs using marine brown alga *Ecklonia cava* and stated that synthesized NPs are spherical and triangular in SEM analysis (Venkatesan et al., 2014). Similarly, Xie et al. synthesized AuNPs using *C. vulgaris *and they showed their triangular and hexagonal-edged structures with FE-SEM analysis (Xie et al., 2007). In also our study, spherical shaped nanoparticles were suggested with both TEM and FE-SEM data. In addition, according to TEM micrographs, triangular nanoparticles were seen in some places.

Microalgae can be considered as a bionanofactory for metal NPs production. However, depending on the algae type chosen and the used procedure, NP synthesis can be carried out either intracellularly or extracellularly. The intrasellular synthesis depends on the metabolism of the used algae; after the metal ions are taken up in a dose-dependent manner by the cell, enzymatically converted into metallic forms. In also extracellular synthesis, metal ions are trapped on the algae cell surface and ions are reduced by enzymes (Li et al., 2011). Different conditions can lead to different sizes and shapes in the synthesis of NPs. The reaction condition can be optimized by changing various experimental factors (Tikariha et al., 2012). Singaravelu et al. performed extracellular gold nanoparticle biosynthesis using *Sargassum wightii* and stated that this synthesis is very stable and provides gold nanoparticle recovery in a short time (Singaravelu et al., 2007). In our study, it is thought that factors such as chemical composition, stability or particle size have been improved by the detailed optimization studies performed in the extracellular synthesis method. The NPs sizes obtained were determined as 5–15 nm as a result of extracellular synthesis and as 20–40 nm as a result of intrasellular synthesis. However, in the intracellular synthesis method, only time-dependent changes were observed. Therefore, the size difference in the results of both synthesis is thought to be due to the change in experimental factors.

Optimization studies in nanoparticle synthesis are very important to obtain most appropriate reaction parameters. In the present study, we investigated the influence of time, pH value, and salt concentration on the extracellular synthesis and the influence of time on the intrasellular synthesis. In the literature, the effects of various environmental factors on algae-mediated AuNPs synthesis have been extensively investigated. Costa et al. synthesized AuNPs using *Sargassum cymosum* algae extract and carried out optimization studies on pH value, stirring rate, temperature, different extract, and tetrachloroauric acid concentrations in order to examine the effects of reaction variables on synthesis. The authors stated that the stirring rate factor did not affect the synthesis and properties of AuNPs. However, the best results were obtained when pH <5, temperature 60–80 °C and when employing an algae extract to metal precursor mass ratio 5–20. The NPs obtained are spherical and of 5–22 nm in size (Costa et al., 2020). In the study of Rajeshkumar et al., AuNPs synthesis was made with marine brown algae *Padina tetrastromatica*. The authors investigated the effects of changes in algae extract concentration, pH value, and temperature parameters on synthesis. In the study, as the algae extract concentration increased, the NPs dimensions also increased. Again, as the pH value increased, an increase in the absorbance density was observed. In the same study, the most ideal conditions have been reported as 7.5 mL algae extract concentration, pH = 6, and temperature = 80 ºC, and AuNPs sizes vary between 8–10 nm (Rajeshkumar et al., 2017). According to the optimization data obtained in our study, 1 mM HauCl4 concentration, pH value of 6 and time 60th minute gave the most ideal results. In a study of Princy et al., AuNPs were obtained by green synthesis mediated by the macroalgae *Padina tetrastromatica*. The effects of seaweed extract amount, temperature, time, precursor metal ion concentration, reaction time, and pH value on reaction conditions were investigated. In this study, the reaction time was monitored for 0 min to 48 h. AuNPs synthesis started at the 5th minute and was completed within 24 h (Princy and Gopinath, 2018). In also our study, the synthesis started at the 10th min and was completed at the 60th min. This difference may be due to the used algae species. Oza et al. stated that AuNPs obtained with *Chlorella pyrenoidusa* are spherically formed at alkaline pH value and anisotropic when pH value is 4, and NPs dimensions are 25–30 nm (Oza et al., 2012). In also our study, AuNPs obtained by intracellular synthesis are 20–40 nm and those obtained by exrasellular synthesis are 5–15 nm and similar to the literature results. 

Precursor salt concentration is a factor that determines the formation of nanoparticles, and when this changes, the SPR band shifts towards different wavelengths. According to TEM data, as the precursor concentration increases, amount of different sized particles increases (Mnisi et al., 2016). In our study, when 0.5 mM HAuCl4 was used, absorbance was determined at 657 nm, and nanoparticle synthesis was not at the desired level. When 5 mM precursor was used, nanoparticle formation was not observed. Using 1 mM HAuCl4 showed that small and dispersed gold nanoparticles were synthesized by giving absorbance at 565 nm. Therefore, the study was continued with 1 mM precursor. By considering these results, the optimum concentration of HAuCl4 for the preparation of AuNPs is 1 mM. Similar interpretations were also reported by previous studies, and they stated that there is agglomeration at concentrations other than 1 mM. (Princy and Gopinath, 2018; Ghosh et al., 2011; Mnisi et al., 2016).

One of the most important factors affecting the production rate, stability and biosynthesis of AuNPs is the pH value. At low pH value, AuNPs agglomerate because there will be less nucleation (pH <5). The stability of nanomaterials is better if the pH value is between 5 and 11. On the other hand, with the increase of pH value, the absorbance value also increases and there is a color change. Nanoparticles are larger at acidic pH values and this is seen with the shift in the SPR spectrum. Therefore, increasing the pH value provides capping of the NPs surface and supports the formation of smaller NPs. In also our study, it was determined that small-sized monodisperse nanoparticles were synthesized at pH 8 (533 nm) in accordance with these data (Khalil et al., 2012; Ahmed and Ikram, 2016).

Determining the organic material content during the synthesis of AuNPs or establishing relationship between the bonds is the basis of these studies. For example, González-Ballesteros et al. emphasized that the total phenolic content of *Cystoseira baccata* marine algae influences green synthesis (González-Ballesteros et al., 2017). Venkatesan et al. reported that alcohols and hydroxil in functional groups were effective at *Ecklonia cava* extract (Venkatesan et al., 2014). In our study, too, extract associated alkenes, carbonyl contents, proteins and especially aromatic compounds were found to be effective by before and after synthesis comparisons in *C. sorokiniana.*

AuNPs may enter the cells depending on size and shape (Chen et al., 2009), and their antimicrobial efficacies are tried to be explained with different mechanisms. NPs disrupt the electrostatic flow across the cell membrane and destroy the membrane; however, this mechanism has not been fully clarified (Abdel-Raouf et al., 2017). AuNPs especially react with sulfur or phosphorus holding bases. When NPs are attached to thiol groups of enzymes, free radicals are formed and the respiratory chain is destroyed, which leads to cell death. According to another hypothesis, AuNPs reduce ATPase activity or they prevent the tRNA from binding to the ribosomal unit unit. Therefore, as the size of NPs increases, their antimicrobial activity decreases. Factors such as the cell wall composition of the microorganism or its surface chemistry also affect the antimicrobial activity (Nadeem et al., 2017).

In a previous study, the antifungal activity of AuNPs obtained using *C. vulgaris* extract against *C. albicans* was evaluated by disk diffusion test (Annamalai and Nallamuthu 2015). The authors used the same concentration of metal salt with our study and stated the obtained zone diameter as 18 mm. Similarly, in the studies of Hassaan and Hosny, AuNPs were obtained by green synthesis mediated by *C. Vulgaris,* and the zone diameter obtained against the same strain was reported as 16 mm (Hassaan and Hosny, 2018). In our study, the antifungal efficiency of AuNPs against *C. albicans* was found to be lower and it was determined as 10 mm. The antimicrobial effectiveness of AuNPs may vary according to many parameters. Concentration, size and shape characteristics, type of microorganism, synthesis method, and incubation time can affect antimicrobial efficacy (Tao, 2018). This difference in zone diameter may be due to use of a different *Chlorella* species. In addition, the size of AuNPs (5-15 nm) obtained in our study is relatively larger than the NPs sizes (2–10 nm) reported by the researchers. This may have also slowed the diffusion of gold nanoparticles through the cell membrane and affected the antifungal activity. When the ultrastructural effects of AuNPs on different *Candida *species are examined by TEM, the most obvious damages were on cell wall and membrane structure. In addition, it has been observed many cells whose integrity has been disrupted after the damage formation in the cytoplasm and nucleus structures. 

## 5. Conclusion

As a result, AuNPs synthesis was carried out by green synthesis with *C. sorokiniana* algae. Nontoxic chemicals were used for synthesis and no harmful waste was left to the environment. The most suitable reaction parameters have been determined with pH value, time, and metal ion concentration optimization studies. C-AuNPs produced showed strong antifungal efficacy and exhibited advanced damage findings on *Candida* cells. Our findings are important for their potentials in pharmaceutical applications. However, detailed research is needed on its safe use in nanomedicine. The significant antifungal activity of the synthesized nanoparticles has been found promising both in the development of alternative antifungal treatment regimens and in combating the problem of drug resistance.
